# S94. INTEGRATED DIABETES MANAGEMENT FOR INDIVIDUALS WITH SERIOUS MENTAL ILLNESS

**DOI:** 10.1093/schbul/sby018.881

**Published:** 2018-04-01

**Authors:** Kristina Cieslak, Corinne Cather, Sarah Maclaurin, Deborah Wexler, Anne Thorndike, Trina Chang, Gladys Pachas, Mike Vilme, Oliver Freudenreich, Anne Eden Evins

**Affiliations:** Massachusetts General Hospital

## Abstract

**Background:**

Premature mortality due to cardiovascular disease in those with schizophrenia is the largest lifespan disparity in the US and is growing; adults in the US with schizophrenia die on average 28 years earlier than those in the general population. An estimated one in five people with severe mental illness (SMI) has diabetes; lifetime rates of diabetes among those with SMI are two to three times higher than for those in the general population. Contributing factors to this astonishingly high rate of diabetes include effects of antipsychotic medication, unhealthy lifestyle, and likely factors related to schizophrenia itself. High rates of tobacco dependence and poor understanding of diabetes management combine to cause to the extraordinarily high morbidity and mortality associated with diabetes in those with SMI. There exists a significant gap in the literature for theory and evidence-based interventions to improve the ability of those with SMI to manage their diabetes.

**Methods:**

We have developed a 16-week tailored behavioral and educational group intervention for individuals with schizophrenia and diabetes, utilizing the concept of ‘reverse integrated care,’ bringing medical intervention into the community mental health setting. Core features of this intervention include motivational interviewing, basic education, and problem-solving. The primary outcome of this study is glycemic control, as measured by hemoglobin A1C (HbA1C). Secondary outcomes include lipid panel, measures of diabetes knowledge and self-management, blood pressure, weight, BMI, and step count.

**Results:**

Thirty individuals were consented and randomized to a two-period crossover design consisting of a 16-week group intervention and a 16-week observation period. Average HbA1c at baseline=7.5, range=5.9–13.4. Seventeen individuals successfully completed the intervention. An average 0.59-point reduction in HbA1c was observed from baseline to the end of the 16-week active intervention (t=1.99, DF=17, p=0.063). A marginally significant weight reduction was observed from baseline to week 16 in the active condition of 5.3 pounds (t=2.07, DF=17, p=0.054). Ten participants lost greater than five pounds. Significant changes were observed in increased average step count of 3189 steps/day (t=2.25, DF=17, p=0.038), and improved scores on diet (t=2.84, DF=17, p=0.01), exercise (t=2.24, DF=17, p=0.039), and foot care (t=2.99, DF=17, p=0.01) diabetes self-care measures. Promising decreases were seen in systolic blood pressure – those with baseline >130 systolic blood pressure reducing from an average of 138 to 125; diastolic blood pressure – those with baseline >90 reduced from an average of 93 to 80; a 10-point average reduction in total cholesterol (t=-1.13, DF=17, p=0.27), and 50-point average reduction in triglycerides (t=-1.29, DF=17, p=0.21). A continued decrease was observed for A1C, weight, and triglycerides in the first active intervention group 16-weeks post-completion, suggesting sustainability of gains made during the intervention.

**Discussion:**

There is a pressing need to address the morbidity and premature mortality related to modifiable health behaviors in this underserved population, yet individuals with SMI and diabetes are much less likely to be identified or to receive recommended diabetes care and monitoring. We hope to further establish and refine a standard of care diabetes education curriculum, tailored for individuals with SMI, a population with high prevalence of diabetes but low rates of diabetes diagnosis, education, and treatment. Results from year one demonstrate this program to be easily implementable, well-accepted, socially relevant and effective.

